# Supplementary material for: Are carbon emissions trading and green financial instruments synergistic? -Comprehensive quantitative research based on content analysis
Source: PLoS One. 2024 Mar 7;19(3):e0298601. doi: 10.1371/journal.pone.0298601 (PMC10919601; doi:10.1371/journal.pone.0298601)
Supplement: S1 Data — (ZIP) [file pone.0298601.s001.zip › Data/地级市Z_F工作报告.zip!/╣≤╓▌/░▓╦│╩╨/└┤╫╘╣1⁄2╓┌║┼í╛┬φ┐╦╩2╛▌═°í┐_2019.docx]

各位代表：

现在，我代表市人民政府向大会作工作报告，请予审议，并请市政协委员和其他列席同志提出意见。

一、2018年工作回顾

过去的一年，我们坚持以习近平新时代中国特色社会主义思想为指导，深入学习贯彻党的十九大精神和习近平总书记对贵州工作系列重要指示批示精神，在市委的坚强领导下，在市人大、市政协的监督支持下，认真贯彻落实省委十二届三次、四次全会精神和市委四届四次全会精神，团结和带领全市各族人民，坚决打好三大攻坚战，深入实施三大战略行动，扎实打好“一二四”攻坚战，全市经济社会实现持续健康稳定发展。

——主要指标稳中向好。预计，全市实现地区生产总值900亿元，增长11%；固定资产投资增长14%；规模以上工业增加值增长11.2%；社会消费品零售总额增长8.1%；一般公共预算收入增长1.4%。全市金融秩序总体稳定，政府债务风险总体可控。

——脱贫攻坚稳扎稳打。春风行动、夏秋攻势成效显著，涌现出“大坝模式”“彩虹模式”等经验和做法。全市减少贫困人口10.88万人，贫困发生率下降到3.7%，180个贫困村出列，西秀区、平坝区通过国家评估验收成功脱贫摘帽。

——基础设施稳固改善。安紫、普织高速建成通车，30户以上村民组100%通硬化路。纳井田、猫洞河水库下闸蓄水。新建10千伏及以下农网线路3500公里。铺设城镇燃气管网75公里。30户以上自然村4G网络覆盖率达80%。

——高质量发展稳健前行。安顺国家农业科技园区顺利通过科技部评估验收。无公害农产品产地认证面积达321.75万亩，有机产品认证达35个，地理标志产品达22个。柳江公司荣获国家科学技术进步奖二等奖，安大公司获省长质量奖提名奖。高技术制造业增加值占规上工业增加值比重达31%，科技进步综合指数达55.9%，单位地区生产总值能耗下降4%。接待游客突破1亿人次、旅游总收入超过1000亿元。

——生活水平稳步提升。城镇和农村居民人均可支配收入29675元和9878元，分别增长9%、10.3%。居民日用品类、家用电器类消费保持两位数增长，体育、教育、文化、养老、医疗等服务消费供需两旺。居民消费价格涨幅控制在2%。

一年来，我们主要做了以下工作：

（一）围绕脱贫攻坚，尽锐出战，全面小康加快推进

脱贫责任扣紧扣牢。我们认真贯彻习近平总书记关于扶贫工作的重要论述和贵州大扶贫战略行动部署，落实“五主五包”责任制，层层立下军令状。市委市政府出台了《关于深入实施打赢脱贫攻坚战三年行动发起总攻夺取全胜的实施意见》。市政府将脱贫攻坚作为常务会议常设议题，加强工作研究和落实。扎实开展扶贫领域“五个专项治理”和巡视问题整改，脱贫攻坚质量稳步提高。先后举行两次市级脱贫攻坚表彰活动，熔铸提振安顺脱贫精气神。

“四场硬仗”打好打赢。我们围绕脱贫攻坚短板和难点，全力打好“四场硬仗”组合拳。投入扶贫子基金24.75亿元实施产业扶贫。完成2017年度易地扶贫搬迁14个安置点3.53万人搬迁，2018年度6个安置点主体工程全部完工。建成“组组通”硬化路1500公里，解决14.6万建档立卡贫困人口的饮水安全问题。资助23136名建档立卡贫困学生6249.97万元，免除12040名普通高中贫困学生学费852.46万元。实现建档立卡贫困人口参合全覆盖，住院费用实际补偿比达90%，实施贫困人口大病集中专项救治行动，免费救治819人次，补偿1883.79万元。完成农村危房改造13740户，竣工率达100%。

“四个聚焦”攻坚克难。我们全力整合各类帮扶资源，千方百计攻克深度贫困堡垒。57名厅级干部、121名县处级干部定点帮扶深度贫困村，选派3808名干部担任第一书记和驻村干部。统筹资金42.9亿元用于深度贫困地区交通、水利、电力等基础设施建设。安排1.78亿元扶贫产业子基金用于178个深度贫困村发展产业，空壳村基本实现“脱壳”。青岛市投入财政资金2.46亿元、社会帮扶资金2549万元对口帮扶我市，青岛鑫源环保、鸿德盛中药饮片、红星工业辣椒等项目落地投产，教育医疗、人才支援、劳务协作等领域不断深化，携手奔小康向纵深发展。航空工业、南方电网、开磷集团等企业投入2.54亿元定点帮扶我市4个贫困县。

（二）围绕项目投资，提质扩容，发展支撑不断夯实

项目带动持续发力。我们紧紧围绕中央、省、市重大发展战略谋划项目，重大项目库亿元以上项目保持在500个以上。全年实施重大工程和重点项目675个，总投资6452.4亿元，完成年度投资1201亿元。先后组织10次集中开工，新开工项目259个。192个项目获中央、省补助资金25.67亿元。51.38亿元全域旅游军民融合扶贫投资基金落地实施。

有效投资持续扩大。我们狠抓关键领域和薄弱环节，着力补短板、增动能。安大航空锻造产业园、黎阳国际军民融合产业基地等产业项目加快建设。安六铁路顺利铺轨，都香、紫望高速建设加快推进，建成普通国省道93公里，改造县乡道56公里，完成交通投资130亿元。黄家湾、打冲沟、白沙水库成功截流，新场河、老营等8座水库主体工程完工，完成水利投资30亿元。完成城建投资330亿元、农网改造升级投资5.8亿元、信息基础设施投资7亿元。

产业大招商成效明显。我们大力实施“千企引进”工程，先后赴深圳、海口、青岛等地开展主题招商，通过黄果树飞行大会、石博会、贵洽会等平台开展招商推介。引进温氏集团、上海电气、江苏中利等落户我市，全年新引进省外投资500万元以上项目684个，新增到位资金905亿元。

（三）围绕高质量发展，优化结构，产业发展提档升级

农业产业成果丰硕。我们紧扣“八要素”，全面开展振兴农村经济的深刻产业革命。全年调减玉米种植面积63.72万亩，蔬菜、茶叶、水果、中药材种植面积分别增长8.1%、13.3%、10.5%、3.9%，收购烤烟10.29万担。关岭牛、金刺梨、蜂糖李、红芯薯、普定韭黄、紫云花猪等特色农产品产业化步伐不断加快。柳江鸡蛋、黔山屯土鸡蛋荣获“贵州省十大优质特色禽产品”称号，畜牧业增加值占农林牧渔业增加值比重连续七年排全省第一。产销对接不断加强，“安货出山”持续推进，订单农产品销售额达13.85亿元。新增市级以上龙头企业26家、农民专业合作社421家，规模以上农产品加工企业达102家。

工业产业强筋健骨。我们着力构建特色鲜明、结构合理、功能配套、竞争力强的工业体系。“千企改造”工程扎实推进，完成投资105亿元，安顺铝业等18户企业纳入省级龙头企业、高成长性企业升级改造。传统支柱产业支撑带动作用明显，煤炭、电力、有色等行业增加值增速高于全市工业平均水平。新兴产业拉动效应突出，全市规模以上电子信息制造业、医药行业、装备制造业增加值分别增长70%、15%、13%。军民融合深度发展，贵飞工业联合体已扩展到6户企业配套生产，贵飞公司自主研制生产的FTC-2000G外贸飞机首飞成功。大数据产业加快发展，“安顺彩云”大数据综合云平台成功上线，安顺煤炭大数据管理平台投入使用。102户企业实现大数据与实体经济深度融合。

现代服务业蓬勃发展。我们以全域旅游为龙头，引领现代服务业提档升级。旅游产品“1+5个100工程”实现投资24.38亿元。小河湾、高荡被评为国家4A级旅游景区，多彩万象城获批省级旅游度假区。我市荣获2018年度《中国国家旅游》最佳全域旅游目的地，再次荣膺“中国最佳避暑旅游城市”。旅游业持续“井喷”，接待游客人次、旅游总收入分别增长35%、37%。成功承办省第四届大健康医药产业发展大会。全市大健康医药产业实现增加值120亿元。电子商务加快发展，整体推进国家级电子商务进农村综合示范县、省级农商互联示范县建设，共获1亿元资金支持。黔中（安顺）物流园区入选第二批国家级示范物流园区。金融服务、家政服务、快递物流等加快发展。实现服务业增加值445亿元，增长12%。

（四）围绕新型城镇化建设，融合发展，城乡面貌显著改观

城镇化进程加快推进。我们全力加快山地特色新型城镇化建设，统筹“城、镇、村”融合发展。在全省率先完成市级多规融合专章规划。积极推广“一分三向”安顺模式，构建了“中心城区—县城和特色镇—美丽乡村”的新型城镇体系，新增城镇人口10万人，户籍人口和常住人口城镇化率分别达41.5%、52%。推广实施“1+N”镇村联动模式，带动278个村“联动、联建、联富、联美”发展。

城市“颜值”显著提升。我们牢牢把握“特、新、化、美”内涵，全力建设“五型城市”。14个城市“双修”试点项目基本完工。打通中心城区横一路等5条断头路，新增城市道路50公里，城市路网不断优化。贯彻落实《安顺市虹山湖保护条例》，娄湖、贯城河焕然一新，城市建成区人均公园绿地面积达20.43平方米。完成棚户区改造3.26万户。治理城区黑臭水体11个，做法和成效得到生态环保部点赞。

城乡活力持续焕发。我们不断加强县城、示范小城镇和美丽乡村建设，西秀、平坝、普定同城化加快推进，镇宁、关岭、紫云县城活力焕发。建成澳维天下乐城等10个城市综合体。打造5个示范小城镇，建成“8+X”项目90个。新增20个美丽乡村，完成866个村庄规划编制。14个少数民族村寨入选贵州省第三批少数民族特色村寨，大坝村获评中国美丽休闲乡村。

（五）围绕绿色发展，守牢底线，生态环境明显好转

生态建设成效显著。我们深入践行绿水青山就是金山银山理念，划定生态红线面积2317.65平方公里，优化确定永久基本农田310.16万亩，综合治理石漠化面积108.63平方公里，治理水土流失面积220平方公里，完成营造林46.13万亩，森林覆盖率达到56%。邢江河国家湿地公园通过国家验收，紫云格凸河、镇宁高荡·夜郎洞分别获国家地质公园、省级地质公园称号，安顺药王谷荣获第四批全国森林康养基地。6个建制县区成功创建省级森林城市。

环境质量大幅改善。我们牢牢守好发展和生态两条底线，全力打好污染防治“五场战役”。淘汰燃煤锅炉27台、165.55蒸吨，全市环境空气质量优良率达98.6%。严格落实“河长制”，全域取缔网箱养鱼，国控、省控地表水断面水质优良率均为100%，8个县级以上饮用水源地水质达标率均为100%，县级以上城市污水处理率达92.5%。工业固体废物综合利用率达76%。城市噪声污染防治持续强化。全面完成中央、省委环保督察反馈问题整改。

（六）围绕改革开放，激发活力，发展动能持续增强

深化改革蹄疾步稳。我们以庆祝改革开放40周年为契机，实现各领域改革向纵深发展。供给侧结构性改革持续推进，为企业降低各类成本40亿元。推进“三权”促“三变”改革，“塘约经验”推广率达90.4%，集体经济超百万元的村达429个。深化商事制度改革，各类市场主体达15万户，民营经济增加值占生产总值比重达65%。完成税务征管体制改革。创新驱动加快发展，新增高新技术企业24户、院士工作站2个、省级科技创新平台3个。

对外开放逐步拓展。我们坚持内引外联开放发展，成功举办黄果树·屯堡自然文化遗产分会、一带一路贵州（安顺)国际商旅陆港投资合作交流会。积极参加数博会、酒博会、进博会等大型活动，与港澳台、欧盟、东盟等地交流合作不断加强。安顺高新区、黄铺新区成为我市开放发展的新高地。全市外贸进出口总额达2.3亿美元，实际利用外资9370万美元。

（七）围绕民生福祉，补齐短板，民生事业稳步发展

社会保障稳步提高。我们坚持以人为本，不断织牢织密民生保障兜底网。城镇新增就业6.5万人，城镇登记失业率为3.2%。城市和农村低保平均标准提高到603元/月、3906元/年，分别增长6.5%、9.4%；保障城乡困难群众12.15万人，发放最低生活保障金4.28亿元。保障特困供养人员4143人，发放救助供养金5785万元。公租房分配率达93%。主城区实现双水源供水，有效解决用水紧张难题。“十件实事”涵盖的17项具体工作，1项基本完成，16项全面完成。

社会事业稳步提升。我们始终坚持把改善民生作为工作的出发点，不断提高公共服务有效供给。完成“1+7”民生工程投资171.53亿元。全市县域义务教育基本均衡顺利通过国家验收。一批优质民办学校落户我市。医疗卫生资源逐步优化，市、县、乡三级远程医疗全部实现互联互通。基层医疗卫生服务能力三年提升计划全面完成。连续七次荣获全国无偿献血先进城市。成功举办第二届全市运动会，紫云格凸贵州攀岩基地成为全国首个授牌的“国家攀岩训练基地”。多彩贵州“广电云”户户用工程达18万户。“全民阅读”等文化活动蓬勃发展。

社会治理稳步推进。我们不断创新和加强社会治理，社会大局和谐稳定。法律服务能力加快提升，实现村（居）法律顾问全覆盖。信访工作责任制度持续完善，安全生产形势持续稳定，食品药品安全监管不断加强，地质灾害防治稳步推进。建成公安大数据中心，群众安全感、满意度分别排名全省第2、第3，创历史最佳成绩。外事侨务、民族宗教、国防教育、人民防空、防灾减灾、档案管理、应急管理、地方志等各项事业取得新进步。工会、共青团、妇联、文联、社科联、残联、侨联、科协、关工委、红十字会、计生协会等群团组织桥梁纽带作用得到充分发挥。

（八）围绕政府建设，提升效能，政务服务高效便捷

我们以中央、省委巡视反馈问题整改为契机，着力加强作风建设，坚持不懈纠治“四风”，全面推进政府系统党风廉政建设。我们坚持依法行政，贯彻实施宪法和监察法，自觉接受法律监督、监察监督、审计监督、民主监督、社会监督和舆论监督，依法办理市人大代表建议59件、市政协提案185件，办复率100%。我们全面推进政务公开，网站在线访谈、民意征集、政府数据开放等工作不断加强。我们深入推进“放管服”改革，公布实施新一轮权力清单和责任清单，行政审批办理时限压缩了56%，在全省率先实现不动产交易登记“一窗式”服务，政务服务更加高效便捷。

回顾过去一年的工作，成绩来之不易！这是省委、省政府和市委坚强领导的结果，是市人大依法监督和市政协民主监督的结果，是全市各族人民团结拼搏的结果。在此，我谨代表市人民政府，向全市各族人民，向人大代表、政协委员，向各民主党派、工商联、无党派人士、人民团体，向驻安人民解放军、武警、消防救援、公安政法干警、中央和省驻安单位，向老同志及关心支持安顺经济社会发展的各界人士，表示衷心的感谢并致以崇高的敬意！

各位代表，在看到成绩的同时，我们也清醒地认识到存在的问题和不足。主要表现在：经济总量小、总体实力弱、提速发展压力大；贫困程度深、攻坚难度大、脱贫任务艰巨；新旧动能转换不足、实体经济质量不高、发展动力支撑不强；传统税源支撑下滑、新兴财源培育不够、财税持续增收难度大；在基本公共服务方面，群众还有一些不满意的地方；形式主义、官僚主义还不同程度存在，政府效能还需进一步提高。我们将努力破解难题，全力做好工作，决不辜负全市人民的重托！

二、2019年工作重点

今年是新中国成立70周年，是决胜全面建成小康社会第一个百年奋斗目标的关键之年，是我市脱贫攻坚决战之年。做好今年的政府工作，要以习近平新时代中国特色社会主义思想为指导，全面贯彻党的十九大和习近平总书记对贵州工作系列重要指示批示精神，统筹推进“五位一体”总体布局和协调推进“四个全面”战略布局，坚持稳中求进工作总基调，坚持新发展理念，坚持推进高质量发展，坚持以供给侧结构性改革为主线，坚持深化市场化改革、扩大高水平开放，进一步做好“六个稳”工作，守好发展和生态两条底线，推进实施三大攻坚战，全力实施三大战略行动，持续打好“一二四”攻坚战，保持经济持续健康发展和社会大局稳定，为全面建成小康社会收官打下决定性基础，以优异成绩庆祝中华人民共和国成立70周年！

今年全市经济社会发展的主要预期目标是：地区生产总值增长10.5%左右，固定资产投资增长16%左右，规模以上工业增加值增长10%左右，社会消费品零售总额增长11.5%左右，一般公共预算收入增长6%左右，城乡居民人均可支配收入分别增长9%以上和10%以上，居民消费价格涨幅控制在3%左右。

围绕实现以上目标，我们将重点抓好九个方面工作。

（一）抓紧抓实脱贫攻坚，着力冲刺全面小康

全面发起脱贫总攻。用好用活绿色产业扶贫投资基金，开展特色产业提升工程，确保每户贫困家庭至少有2个增收产业。扎实推进石漠化片区水利精准扶贫示范区建设，全面解决农村人口饮水安全问题。完成2018年度易地扶贫搬迁6个安置点搬迁入住，加强和完善易地扶贫搬迁后续扶持，加快搬迁群众市民化步伐。全面落实教育精准扶贫资助政策。深入实施健康扶贫，贫困人口住院费用实际补偿比稳定保持在90%以上。推进农村危房改造，有效解决贫困户住房安全问题。持续开展“五个专项治理”，切实提升扶贫质量。扎实推进东西部扶贫协作，积极引导企业帮扶和社会帮扶。深化“四个聚焦”，坚持扶贫与扶志、扶智相结合，全力抓好紫云县，简嘎乡、大营镇和178个深度贫困村脱贫攻坚。持续巩固西秀区、平坝区脱贫成效，实现普定县、镇宁县通过评估验收脱贫摘帽和全市110个贫困村出列，关岭县达到退出标准。

全面提升产业成效。切实抓好粮食生产，合理调整“粮经饲”结构，增加优质绿色农产品。加快建设全省唯一的高标准蔬菜保供基地。认真落实“八要素”要求，以500亩以上坝区为重点推进农业产业结构调整，加快发展蔬菜、茶叶、烤烟、中药材等高效经济作物及金刺梨、蜂糖李、火龙果、红芯薯等特色农产品。鼓励发展林下经济，大力发展生态畜禽、淡水生态养殖。加强农村土地整治和农田水利基础设施建设，建成高标准农田12.68万亩。推进农产品精深加工，扎实抓好产销对接，大力发展订单生产，加快冷链物流体系建设和电商扶贫。继续推广“塘约经验”“大坝模式”“一村一公司”“菜单式扶贫”等做法，不断创新利益联结机制。培育壮大新型农业经营主体，新增省级龙头企业6家、市级龙头企业17家、农民专业合作社400家。一产增加值增长6.5%左右。

全面推进乡村振兴。编制实施《安顺市乡村振兴战略规划》。加强脱贫攻坚与乡村振兴统筹衔接，做优做强农村产业。推进“四在农家·美丽乡村”小康升级行动计划和田园综合体建设。继续实施“厕所革命”，加强农村生活垃圾治理，推动城镇污水管网向乡村延伸，逐步实现污水集中收集处理。实施农村公共服务提升计划，引导科技、教育、文化、医疗、卫生、体育、法律等向乡村延伸覆盖。严厉整治乡村滥办酒席，遏制各类陈规陋习。加强农村群众性自治组织建设，完善现代乡村社会治理体系。改造和治理“空心村”，增强乡村活力。实施新型职业农民培育工程，培训农村青壮年劳动力3万人。

（二）抓紧抓实污染防治，着力建设美丽安顺

加强生态建设。严格环保准入，制定“三线一单”，全面排查违法违规挤占生态空间、破坏自然遗迹等行为。全面完成第三次国土调查。加强天然林保护，完成营造林81万亩，森林覆盖率提高到58%。综合治理石漠化面积120平方公里，治理水土流失面积220平方公里。加快推动紫云县黑颈长尾雉自然保护区科考和申报工作，推动普定夜郎湖、镇宁桫椤河、紫云翠河申建省级湿地公园。加快国家生态文明试验区（安顺）各项任务建设，创建国家森林城市。

加大环境治理。打好蓝天保卫战，淘汰重点区域20蒸吨及以下燃煤设施，依法取缔城区周边非法采矿、采石和采砂企业，严控农村秸秆焚烧，全市环境空气质量优良率达到95%以上。打好碧水保卫战，认真落实“河长制”，抓实县级及以上集中式饮用水水源地保护工作，完成全市90%的黑臭水体整治，加强农业面源污染防治和畜禽养殖废弃物资源化利用，县级以上城市污水处理率达到93%以上。打好净土保卫战，编制完成农用地土壤环境质量分类清单。打好固废治理攻坚战，提升固废资源综合利用率。

加快绿色发展。深入实施绿色制造三年专项行动计划，大力发展绿色经济“四型产业”，推动园区集聚绿色工厂，促进产业延长绿色链条，创建绿色示范园区3个以上。实施森林扩面提质增效三年行动计划，林业产值达200亿元。深化矿业权出让制度改革，创建省级绿色矿业发展示范区。加强重点用能和重点排放单位监管，能源消费增量、单位地区生产总值能耗、化学需氧量、氨氮排放量等指标控制在省下达标准范围内。

（三）抓紧抓实风险防范，着力维护社会稳定

切实防范化解金融债务风险。严格落实地方金融监管责任，开展各类新兴金融业态风险排查治理，严厉打击非法集资，确保不发生区域性金融风险。严格落实政府债务管理相关规定，健全“借用还”与“责权利”相统一的地方政府性债务管理机制，强化市、县两级政府债务限额管理和预算管理，用好债务风险应急资金池，有效防范和化解政府性债务风险。多途径盘活存量资产，着力涵养和培植财源。

切实防范化解社会安全风险。加强安全生产“双控”体系建设，强化道路运输、建筑施工、矿山、危险化学品、地质灾害等隐患排查治理，加强食品药品全链条监管，规范餐厨废弃物管理，坚决遏制重特大安全事故发生。健全完善信访工作责任制度，依法分类处理各类信访诉求。完善应急管理体系，提高突发事件快速响应和应急处置能力。深入开展公共安全大整治、扫黑除恶专项斗争。创新发展新时代“枫桥经验”，加快推进基层社会治理现代化，让社会更加公平、有序、和谐。

（四）抓紧抓实有效投资，着力增强发展后劲

做实项目基础。围绕交通、水利、能源、生态环保、城镇基础设施、产业发展等领域谋划和储备项目，确保重大项目库储备亿元项目规模保持在3000亿元以上。加大项目申报力度，争取国家、省资金30亿元以上，增长15%以上。全年实施重大工程和重点项目600个以上，总投资3700亿元以上，年度计划投资800亿元以上。扎实做好项目前期工作，不断强化项目建设要素保障。

加快项目建设。加快推进县域通用航空机场建设前期工作。建成安六城际铁路。开工建设六枝经关岭至安龙高速，加快建设都香高速，建成紫望高速。加快建设贵安大道（安顺经开区至黄果树段），加强普通国省道、县乡道改造，稳步推进乡村窄路面加宽改造。启动建设引子渡、小兴浪航运工程。加快黄家湾、白沙、打冲沟、木桥等水库建设，建成新场河、老营、三岔河等水库。推进城乡信息基础设施建设，实施新一轮农村电网改造升级工程。

聚力招大引强。持续开展产业大招商行动，聚焦军民融合、通用航空、电子信息、大健康医药、文化旅游等产业，策划和编制600个招商项目。强化招商引资责任制，完善项目退出机制，规范招商引资行为。加大项目推介力度，深入开展“青企入安”招商行动和安顺高新区、黄铺新区及黄果树国家路游公园、城镇建设领域等专题招商活动。紧盯500强企业、上市公司和行业龙头，引进一批高端型、配套型、基地型、龙头型产业项目，全年引进省外投资500万元以上项目748个，新增到位资金1000亿元以上。

（五）抓紧抓实新型工业，着力振兴实体经济

壮大产业规模。加大产业园区转型升级，盘活闲置土地，引导关联企业向园区集聚，不断强链补链增链，努力培育壮大航空、智能终端、特色轻工、原材料、能源和战略性新兴产业。紧盯发达地区优势资源，积极发展“飞地经济”。持续推动军民融合发展，加快贵飞工业联合体签约项目落地和风雷公司整体搬迁，推进安大航空锻造产业园、安吉精铸航空产业园及黎阳国际军民融合产业基地等项目建设。推动大数据产业发展，建设“大数据指挥调度中心”和“大数据共享交换平台”，全市规模以上电子信息制造业、软件和信息技术服务业增加值分别增长20%、25%。

做优产业质量。深入实施“千企改造”工程，推动制造业高质量发展。加快推进安铝等量置换项目，延长铝精深加工产业链，推进宏盛化工、红星化工等企业技改扩能，引导贵飞、安吉、华兴科技等企业提升关键产品技术水平，完成投资75亿元以上。持续开展“万企融合”大行动，打造融合标杆项目8个、融合示范项目80个、带动融合企业120户。大力实施质量品牌提升工程，引导产业增品种、提品质、创品牌。

支持企业发展。落实好领导干部联系重点企业联席制度，加大对企业的支持力度。指导企业申报中央建设专项基金、省级工业和信息化专项资金、省工业及省属国有企业绿色发展基金，用好“贵工贷”“贵园信贷通”等政策。完善煤电铝化联动运行协调机制，电煤供应量达到400万吨，推动电力直接交易，降低企业用电成本。加快创新驱动发展，培育省级以上科技型企业80户，建设工程技术中心3个、科技创新战略联盟1个，新增高新技术企业10户。加大三力制药、亿丰实业、汇景纸业、黄果树旅游集团上市培育工作。新增规模以上工业企业35户。

（六）抓紧抓实现代服务业，着力扩大有效消费

推动旅游业创新发展。实施旅游精品景区提升工程、旅游景区培育工程，创建国家全域旅游示范区、贵州旅游名城。挖掘城市旅游热点，加快历史文化街区改造提升，丰富万象旅游城、苗岭屯堡、神骏生态文旅小镇等旅游业态。加快建设黄果树国家路游公园，提档升级关岭古生物化石群国家地质公园。建设一批休闲度假酒店，培育一批生态农庄、森林人家、乡村民宿、青年旅馆，创新生产一批旅游文创商品。支持西秀区办好第六届安顺旅游产业发展大会。加强旅游市场营销，积极开拓海内外旅游市场。全年接待游客人次增长25%，旅游总收入增长30%。

推动服务业繁荣发展。加快发展现代商贸、现代仓储、检验检测、智慧物流等生产性服务业，大力发展文化服务、养老服务、教育服务、体育服务、居民和家庭服务等生活性服务业。加快黔中（安顺）物流园区、中国（安顺）石材城等服务业集聚区发展，新增1个以上省级现代服务业集聚区。推动传统商业综合体创新升级，促进实体零售转型发展和线上线下融合发展。服务业增加值增长12%左右。

推动居民消费扩大升级。大力促进实物消费提档升级，全面取消二手车限迁政策，落实新能源汽车免征车辆购置税、购置补贴等财税优惠政策，促进汽车消费优化升级。大力推进服务消费提质扩容，发展文化旅游体育消费、健康养老家政消费、教育培训托幼消费。推动发展会展消费，支持本地会展企业发展壮大。大力引导消费新模式，积极培育体验消费、智能消费、时尚消费等新热点。大力推动农村消费梯次升级，鼓励农村居民增加家装、家具、家电及大宗商品消费，不断激发居民消费潜力。

（七）抓紧抓实“新型城镇化+”，着力统筹城乡融合

深化综合试点。继续推广“一分三向”安顺模式，落实好户籍制度改革配套政策，户籍人口和常住人口城镇化率分别达到42.5%、53.5%。完善多元化可持续的城镇化投融资机制。继续推广“1+N”镇村联动模式，推进山地特色新型城镇化示范区建设。加快推进“多规合一”，完善城市控制性详规和新一轮城市设计。新开工建设城市地下综合管廊8公里。城市“双修”试点工作顺利通过住建部中期检查验收。

加快城镇建设。积极推进黄铺新区、安顺高铁新城建设，启动北部新城规划建设。深入实施棚改大会战。推进老旧院落改造，启动老旧小区加装电梯试点工作。力争启动主城区南环线建设和安顺东出口立交化改造项目。推进城镇燃气基础设施建设，镇宁县、紫云县开通管道燃气。加大城市环境噪声污染综合整治，用绣花功夫抓好城市治理。支持普定县办好第九届安顺市新型城镇化推进暨小城镇建设发展大会。

促进融合发展。加快贵阳—安顺都市圈建设，稳定增强西秀、平坝、安顺经开区作为中心城区增长极功能，持续推进普定、镇宁、关岭、紫云县域城市“提质培优”发展，深入提升黄果树旅游区的产城融合水平，不断优化区域发展的联动性、协调性。强化产业支撑，加大蔡官、乐平、白岩、丁旗、永宁、猫营、幺铺、白水等城镇的产城融合，新增5个示范小城镇，形成“中心城区集聚、县域城市崛起、城镇产城融合”的区域发展格局。

（八）抓紧抓实改革开放，着力释放发展活力

坚定不移全面深化改革。继续推进供给侧结构性改革，坚决落实减税降费政策和降成本措施，增强微观主体活力。深入推进“三权”促“三变”改革，实现“塘约经验”全市行政村全覆盖。深入开展农村房地一体不动产确权登记，积极探索农村宅基地“三权分置”。深化国有企业改革，促进国有企业实体化、市场化转型和集团化、差别化发展。推进城区公交体制改革。深化统计、供销、教育、医疗、科技等领域改革。

坚定不移扩大对外开放。实施开放带动战略，主动融入“一带一路”和长江经济带，深入推进贵州内陆开放型经济试验区建设。加快安顺高新区、安顺青岛产业园、黄铺新区国际商旅陆港建设。推进各类开发区改革创新，促进产业集聚发展。办好国际半程马拉松、坝陵河国际跳伞、格凸河国际攀岩等活动，用好黄果树飞行大会、国际石博会等平台，不断扩大开放水平。积极引进金融机构在安顺设立分支机构。引导和支持现有外贸企业发展壮大，全年完成外贸进出口总额2.5亿美元，实际利用外资1亿美元以上。

坚定不移发展民营经济。坚持“两个毫不动摇、三个没有改变”，坚决把中央、省、市关于民营经济发展的政策措施落实到位。搭建政银企合作平台，拓宽民营企业融资途径，充分发挥“中小企业应急周转金”应急救助作用，帮助民营企业解决融资难融资贵问题。加大民营企业降本减负力度。加快完善公平竞争的市场环境，在审批许可、经营运行、招投标、资源要素配置等方面对民营企业一视同仁。构建亲清政商关系，深入开展民营企业大走访，以实际行动让民营企业家放心、舒心。民营经济增加值占生产总值比重达到65.1%以上。

（九）抓紧抓实社会事业，着力改善人民生活

强化社会保障。落实积极的创业就业政策，实现城镇新增就业5.8万人，新增创业3000户以上，城镇登记失业率控制在4.2%以内。大力实施“雁归兴安”“锦绣计划”，引导农民工返乡就业创业1.42万人。实施大学生就业创业促进计划和基层成长计划，加强化解过剩产能职工和退伍军人等重点群体就业创业援助。完善城乡医保基金统筹保障机制，落实特殊困难群体筹资参保政策。健全农村留守儿童和妇女、老年人、残疾人等关爱服务体系。加强社会救助、社会福利等工作，推进志愿服务、慈善事业加快发展。

提升公共服务。优先发展教育事业，推动学前教育普惠健康发展，促进义务教育城乡一体化发展。实施优质学校培育计划。推进普通高中优质特色发展和职业教育内涵建设。加大安顺学院“双一流”项目建设，支持安顺职院加快发展。推进健康安顺建设，全面实施14项基本公共卫生服务惠民工程，提升基本公共卫生服务质量。加强重点专科（学科）、智慧医疗建设，提升中医服务能力。加强基层医疗卫生队伍建设。实施“苗圃工程”，推进0—3岁儿童早期综合发展。大力发展养老事业，满足多元化养老服务需求。推出音乐、舞蹈、戏剧、曲艺等节目冲刺国家第十八届群星奖。举办全市第二届文艺调演。加快推进黄果树·屯堡申报世界自然与文化双遗产工作。大力发展竞技体育、群众体育。深入开展全国文明城市、双拥模范城市、禁毒示范城市等创建工作，巩固国家卫生城市创建成果。大力提升公民科学素质，打造紫云猫营科普惠民小镇。开展第四次全国经济普查。加强人民防空、国防后备力量建设。支持工会、共青团、妇联、文联、社科联、残联、侨联、科协、关工委、红十字会、计生协会等依法按章履行职责。进一步做好国防教育、民族宗教、新闻出版、广播影视、地方志、文物、档案、外事、对台、气象等工作。

办好“十件实事”。①减少农村扶贫对象6.76万人；完成易地扶贫搬迁18695人，其中贫困人口14861人。②解决6万人农村饮水安全问题。③新建、改扩建公办幼儿园8所，中小学校16所，新增学位3600个。④实现全市所有乡镇卫生院中医馆（国医馆）全覆盖；建成市公共卫生服务中心、康养中心老年养护楼、市级健康管理中心一期。⑤实施农村老旧房屋透风漏雨问题整治3000户。⑥新建农民工综合劳务市场7个。⑦实施贫困地区生鲜农产品直通车进社区，新建10个生鲜超市（生鲜店）。⑧新增主城区非占道公共停车位1000个。⑨新增城市公交线路3条、新能源公交车90辆。⑩完成村级农体工程30个、乡镇农体工程3个、全民健身路径工程10个。

三、切实加强政府自身建设

各位代表，我们将持续加强政府自身建设，全力为人民群众提供优质高效的政务服务！

一是加强政治建设。坚持把政治建设放在首位，树牢“四个意识”、坚定“四个自信”、践行“两个维护”，坚决落实党中央国务院、省委省政府和市委各项决策部署。坚定理想信念，强化理论武装，严明政治纪律和政治规矩，坚决杜绝“七个有之”，坚决做到“五个必须”。深入开展“不忘初心、牢记使命”主题教育活动，继续推进“两学一做”学习教育常态化制度化。强化中央、省委巡视整改落实，扎实做好巡视“后半篇文章”。

二是严格依法行政。全面贯彻落实依法治国方略，按照法定权限和程序行使权力、履行职责。完善重大事项公众参与、专家论证等制度，推进合法性审查全覆盖。深入推进政务公开，让权力在阳光下运行。加强诚信建设，提高政府公信力。完成《安顺市城市综合执法管理条例》起草工作。自觉接受人大法律监督、工作监督和政协民主监督，主动接受监察监督、审计监督、社会和舆论监督，认真听取人大代表、政协委员、民主党派、工商联、无党派人士和各人民团体意见，做到有权必有责、用权受监督。

三是提升政务效能。完成市县政府机构改革。深入推进“放管服”改革和商事制度改革，优化行政审批流程，严查取消和下放事项“留尾巴”现象。实行市场准入负面清单制度，推动“非禁即入”全面落实。积极推进“双随机、一公开”，推进跨部门联合监管，做到“一次检查、全面体检”，切实提升监管实效。深入推进“互联网+政务服务”，加强市县乡村政务服务平台标准化建设。大力实施“一网通办”，让企业和群众办事更加便捷。

四是坚持廉洁从政。切实履行政府系统全面从严治党主体责任和“一岗双责”，严格执行《中国共产党廉洁自律准则》和《中国共产党纪律处分条例》。贯彻落实中央八项规定及其实施细则精神、省委和市委相关规定，坚持不懈纠治“四风”，坚决破除形式主义、官僚主义。加强重点领域和关键环节审计，严防严控重点工程和重点领域廉政风险。改进和规范督查工作，严厉整治不担当、不作为、慢作为、假作为，全力营造清正廉洁、务实担当的干事创业环境。

各位代表，大道至简、实干为要！让我们更加紧密地团结在以习近平同志为核心的党中央周围，在市委的坚强领导下，紧紧依靠全市各族人民，以坚如磐石的信心、只争朝夕的劲头、坚韧不拔的毅力，奋力续写新时代富美安顺高质量发展新篇章，以更优异的成绩向新中国成立70周年献礼!
